# Combating browning: mechanisms and management strategies in *in vitro* culture of economic woody plants

**DOI:** 10.48130/forres-0024-0026

**Published:** 2024-09-26

**Authors:** Chen Liu, Hongrui Fan, Jiaqi Zhang, Jianing Wu, Mingbing Zhou, Fuliang Cao, Guiyun Tao, Xiaohong Zhou

**Affiliations:** 1 State Key Laboratory of Subtropical Silviculture, Zhejiang A&F University, Lin'an, Hangzhou 311300, Zhejiang, China; 2 State Key Laboratory of Subtropical Silviculture, Bamboo Industry Institute, Zhejiang A&F University, Hangzhou 311300, Zhejiang, China; 3 Co-Innovation Center for Sustainable Forestry in Southern China, College of Forestry, Nanjing Forestry University, Nanjing 210037, China; 4 State Key Laboratory of Subtropical Silviculture, Bamboo Industry Institute , Zhejiang A&F University, Lin'an, Hangzhou 311300, Zhejiang, China

**Keywords:** Browning, *In vitro* culture, Woody plants, Regeneration

## Abstract

Browning presents a significant challenge in the *in vitro* culture of economically important woody plants, primarily due to high levels of lignification and the accumulation of secondary metabolites. This phenomenon hampers the development of efficient regeneration and genetic transformation systems across diverse species. This review examines the internal and external factors contributing to browning, including genetic attributes, tree genotypes, physiological state of explants, explant surface sterilization, medium composition, and overall culture conditions. It explores the underlying mechanisms of browning, particularly enzymatic browning caused by the oxidation of phenolic compounds, and highlights the crucial role of redox pathways and phenolic metabolism. Conventional methods for assessing browning, such as sensory evaluation by researchers and the examination of paraffin sections stained with toluidine blue, are commonly used but introduce significant delays and potential biases. The review emphasizes the importance of accurate and timely browning assessment methods, notably the use of Fluorescein diacetate (FDA) staining, as a reliable and quantitative measure of cell viability to better evaluate browning intensity and progression. Additionally, this review explores the potential manipulation of key genes in the phenylpropanoid pathway to lower phenolic biosynthesis. Advanced strategies, such as regenerative gene manipulation and natural product encapsulation, are also discussed for their potential to improve regeneration outcomes. By integrating recent advancements in molecular biology and tissue culture techniques, this review offers novel insights and potential solutions for mitigating browning, thereby enhancing the regeneration capacities of woody plants. This comprehensive approach addresses the mechanistic bases of browning and underscores the importance of optimizing cultural practices and genetic strategies to overcome this challenge.

## Introduction

The technique of *in vitro* culture is critical in forestry, agriculture, and horticulture, offering significant advantages for plant propagation, precise genetic engineering, and conservation. However, the *in vitro* propagation of woody and medical plants often encounters challenges of browning, primarily caused by high levels of secondary metabolites such as lignin, tannins, and other phenolic compounds. Browning leads to discoloration and necrosis of explants or callus, ultimately impeding their growth and causing cell death. Recognized as one of the three major challenges in plant tissue culture, browning frequently occurs at various stages such as explant induction^[1]^, tissue healing^[2]^, suspension cell culture^[3]^, and protoplast culture^[4]^. Addressing browning is therefore crucial for the successful *in vitro* propagation of these valuable plants.

This review comprehensively examines the recent advancements in understanding browning in the *in vitro* cultures of economically important woody plants, including its mechanisms and potential solutions. It analyzes the primary factors influencing browning, which could be categorized into internal factors and external factors. Additionally, this review examines the browning assessment methods, delves into the current understanding of the mechanisms underlying browning, and discusses potential strategies for its mitigation.

## Factors affecting browning of woody plant *in vitro* culture

Browning *in vitro* culture is influenced by a complex interplay of both internal and external factors. Internal factors primarily include genetic characteristics, such as species and genotypes of tree species, and the physiological status of explants. External factors contain explant treatment, nutrient ingredients, growth regulators, and environmental conditions.

### Internal factors: genetic characteristics and physiological state

#### Species and genotypes

Geographic and species-specific isolation contributes to the diverse characteristics observed in plant populations, including their susceptibility to browning. The susceptibility varies significantly among different plant species and genotypes, largely due to the differing levels of phenolic compounds. For instance, species with higher inherent phenolic content are more prone to browning, making it crucial to select appropriate genotypes for tissue culture. Liu et al.^[5]^ demonstrated a positive correlation between the degree of browning in tissue-cultured pear seedlings and the concentration of soluble phenols across different species; however, no significant correlation was observed with insoluble phenols. Duan & Guo^[6]^ revealed that embryogenic tissues of tangerines, which have higher levels of polyphenols are more susceptible to browning compared to grapefruit and sweet oranges. Similar patterns were also observed in other species such as in litchi (*Litchi chinensis*)^[7]^, lotus (*Nelumbo nucifera*)^[8]^, lemon (*Citrus* × *jambhiri*)^[9]^, and various Magnoliaceae species^[10]^. These findings collectively illustrate the complex interplay between polyphenolic content and genetic predisposition towards browning, emphasizing the necessity of genotype selection for tissue culture.

#### Explants selection and treatment

The selection of explants, including their developmental stage, tissue type, and physiological condition play a critical role in browning. Young, actively growing tissues are generally less susceptible to browning compared to older, more lignified tissues, and they may responsd differently to tissue culture conditions. For example, 7-day-old leaf explants of red raspberry (*Rubus idaeus*) show higher regeneration frequency than 14-day-old explants^[11]^. Furthermore, different parts of the same plant, serving distinct functions and containing different levels of enzymes, hormones, and free amino acids, exhibit distinct metabolic, physiological, and biochemical characteristics that contribute to varying degrees of browning during callus initiation^[12]^. Thus, it is crucial to experiment with different tissues to identify the plant parts with the least susceptibility to browning. Additionally, mechanical damage such as wounds can disrupt the physical distance between phenolic substrate and the oxidase, leading to oxidative reactions^[13]^. Therefore, selecting an appropriate explant and incision size can help reduce the occurrence of browning^[14]^.

Browning in explants can be effectively mitigated through specific pretreatment methods. For example, pretreating explants with NaCl solution has proven effective in suppressing browning in peony (*Paeonia lactiflora* Pall.)^[15]^. The choice of surface sterilization protocol also significantly impacts the physical state of explants and influences the success rate of culture initiation. For instance, explants from *Spartium junceum* L., treated with ethanol for 30 s and sodium hypochlorite for 10 min, show the lowest rate of browning^[16]^. Similarly, Tarinejad^[17]^ observed that explants of walnut yield the optimal surface sterilization results when disinfected using 70% ethanol for 2 min, followed by 5% sodium hypochlorite for 20 min, and 0.7 g/L mercury chloride for 3 min. These cases highlight the importance of developing tailored explant treatment strategies based on specific plant characteristics and requirements. While the inherent properties of the material play a crucial role in browning, it is important to consider that external cultural conditions also significantly impact explant browning.

### External factors: medium composition and culture conditions

#### Basal salt

The nutrient composition of the culture medium significantly impacts both the growth and browning of plant materials *in vitro*. Different salts can influence the ionic balance and osmotic pressure, thereby affecting cell metabolism and viability. Excessive concentrations of inorganic salts can exacerbate the degree of browning in explants by promoting the oxidation of phenolic compounds. For woody plant species, media with lower concentration of basal salts are typically favored. Commonly utilized formulations include 1/2MS (Murashige and Skoog Medium), B5 (Gamborg B5 Medium), WPM (Woody Plant Medium), and DCR (Gupta and Durzan Medium). A comparative study on walnut revealed that DKW (Driver and Kuniyuki Walnut Medium) not only supports notably higher germination and seedling regeneration rates compared to WPM and MS, but also facilitates faster germination, and promotes more robust and greener seedling growth^[18]^. Nitrogen salts in MS medium significantly influence leaf and axillary shoot formation in *Magnolia* × *soulangiana*, as well as phenolic content^[19]^. An excessive concentration of inorganic salts in the medium can lead to phenolic spillover, resulting in the browning of the explants^[20]^. Conversely, reducing the concentration of inorganic salts like NaCl has been shown to mitigate browning in jatropha (*Jatropha curcas*)^[21]^ and apple^[22]^. Similarly, explants of *Cyclobalanopsis chungii* experience less browning rate when cultured on a 1/4 MS medium compared to a normal MS medium^[23]^. Furthermore, substituting nitrate with ammonium salt, which is more heat-stable, decreases the browning mortality rate of bamboo (*Phyllostachys edulis*) callus to 1.2%^[24]^. Feng et al.^[25]^ also found that elevated nitrate concentrations in Ginkgo callus led to severe browning.

#### Carbon source

Carbon source (sugar), as a principal component of tissue culture medium, is essential for cell proliferation and differentiation^[26]^. Also, serving as an osmotic agent, sugar plays a unique role in callus induction and regeneration by affecting the physiology and growth of callus^[27]^. Variations in sugar content can alter the development and morphology of callus by modifying secondary metabolites and related genes^[28,29]^. Optimal sugar concentrations and specific carbon sources can markedly enhance callus induction and regeneration capabilities^[30]^. However, high sugar concentrations can worsen browning and even cause the death of callus^[31]^. Additionally, the browning of explants is also influenced by the ratios of sugar composition. For instance, a specific blend of glucose (5 g/L), sucrose (5 g/L), and fructose (10 g/L) has been shown to effectively control browning in *Taxus brevifolia*^[32]^.

#### Plant growth regulators (PGRs)

PGRs such as auxins and cytokinins play a vital role in cell division and differentiation. Common PGRs utilized for regeneration include indole acetic acid (IAA), indole butyric acid (IBA), 2,4-dichlorophenoxyacetic acid (2,4-D), naphthalene acetic acid (NAA), 6-benzyl adenine (6-BA), thiadiazole phenyl urea (TDZ), and kinetin (KT). Cytokinins like 6-BA and KT enhance polyphenol oxidase (PPO) activity and phenolic compound biosynthesis. Elevated concentrations of 6-BA have been associated with increased browning^[33]^; however, auxins like 2,4-D can delay polyphenol synthesis and reduce browning^[34]^. The combination, concentration, and ratio of PGRs can markedly affect browning in various species. For example, adding 10 mg/L 2,4-D induces callus browning in *Dendrobium officinale*^[35]^. The applications of 6-BA and IBA intensify browning in oil palm^[36]^, while specific concentrations of 6-BA and KT reduce callus browning in *Onobrychis viciaefolia*^[37]^. In cotton, successful plant regeneration has been achieved by supplementing MS medium with appropriate amounts of BA, ZT, or NAA, whereas combinations of 2,4-D, ZT, and NAA lead to browning and plant death^[38]^. Experimentation with different hormone balances can identify optimal conditions to mitigate browning for each species, though this approach requires significant investment of labor and resources.

#### Culture conditions

The phase and pH of the culture medium, and environmental conditions are critical factors influencing browning. The hardness of the medium has an impact on the diffusion rate of phenolic substances. Within a specific range, increasing agar concentration elevates medium hardness and reduces browning^[33]^. For instance, liquid media typically result in less browning compared to solid media with the same composition, as observed in hazelnut (*Corylus avellana* L.) tissue culture^[39]^. pH levels affect the osmotic pressure of the medium and impact the binding sites of phenolics and oxidative enzymes. Generally, pH levels below 5.0 increase susceptibility to browning^[40]^. Environmental factors such as light and temperature also play crucial roles. For instance, light exposure has been linked to increased callus browning severity in *Stellaria dichotoma*, particularly under illumination levels of 8,000, 10,000, and 16,700 lx^[41]^. Furthermore, optimal temperature control is crucial for effectively controlling browning. Maintaining temperatures between 15 and 20 °C has been shown to effectively control the browning rate of young stem tips to less than 10% in Ginkgo^[42]^.

## Mechanisms of browning

To optimize culture conditions and medium formulations, recent research has delved deeply into the mechanisms of browning, to identify strategies to mitigate or prevent browning, improve the preservation and propagation techniques, and expand the range of plant biotechnology applications.

### Non-enzymatic and enzymatic browning in plant* in vitro* culture

Browning during *in vitro* culture can be categorized into non-enzymatic browning and enzymatic browning. Non-enzymatic browning occurs through processes such as the Maillard reaction and sugar pyrolysis, and is not associated with the accumulation of phenolic compounds^[43]^. This type of browning can often be alleviated by successive subculturing and generally does not impede the normal growth and development of the cultured materials. Enzymatic browning, on the other hand, involves the oxidation of phenolics by enzymes such as PPO and peroxidase (POD), leading to the formation of brown quinones that inhibit cell growth and proliferation. This form of browning, which is predominant in plant tissue culture, presents a significant challenge.

Typically, phenolics are stored in the vesicles, while oxidative enzymes are located in the cytoplasm. This compartmentalized distribution serves to prevent oxidative reactions under normal conditions^[44]^. However, stress or damage to plant tissues can disrupt this cellular arrangement, causing contact and reaction of substrate and enzyme, thereby resulting in the production of quinones^[45]^. These quinones further dehydrate and polymerize into black-brown substances, hindering the normal growth of the material. For instance, guava (*Psidium guajava* L.) experienced browning or blackening of explants during *in vitro* culture due to the leaching of phenolics^[46]^. Nevertheless, the propensity for browning varies significantly among different plants, influenced by their phenolic species and content generated within their enzymatic browning pathway.

### Pathways involved in enzymatic browning *in vitro* culture

#### Oxidative reaction

The accumulation of phenolics makes woody plants more prone to browning due to oxidative reactions. PPO, a member of the oxidoreductase family, plays a central role in this process through its two main activities: hydroxylating monophenols, and oxidizing *o*-diphenols to *o*-quinones^[47]^. PPO is classified into three distinct types—tyrosinases, catechol oxidases, and laccases—based on their specific substrates and mechanisms of action^[48]^. The involvement of PPO in enzymatic browning has been extensively studied (Table 1). In Scots pine, callus induced from shoots shows significant browning and increases PPO levels after 2 weeks compared to unbrowned materials^[49]^. Similarly, high PPO activity of callus in Virginia pine (*Pinus virginiana*) leads to browning^[50]^. In walnut, the *JrPPO2* substantially influences browning across different explants^[51]^. Although oxidative reactions mediated by PPO are the direct cause of browning *in vitro*, it is worth noting that under various abiotic stresses, the burst of reactive oxygen species (ROS) can disrupt lipid peroxidation and organelle envelope cytoarchitecture, break the homeostasis of phenols and oxidase, and indirectly affect the occurrence of browning^[52,53]^.

**Table 1:** Genes related to browning in economically important woody plants.

Pathway	Gene	Species	Function	Ref.
Oxidative reaction	*LAC*	*L. chinensis*	Overexpression *LcLAC* promotes callus browning and is involved in polyphenols polymerization.	[7]
	*LAC7*	*Malus domestica*	*MdWRKY31* binds the promoter of *MdLAC7*, positively regulating its activity to promote peel browning.	[58]
	*POD*	*E. urophylla*	PUB and 6-BA enhance POD activity, alleviating browning.	[49]
	*PPO2*	*Juglans regia*	*JrPPO2* shows high activity in browning calluses.	[51]
Phenylpropanoid pathway	*DFRa*	*Camellia sinensis*	*CsDFRa* is involved in the regulation of metabolic flux affecting secondary metabolism and phenotypic characteristics.	[59]
	*FLS*, *UGT*	*Ginkgo biloba* L.	Expression of *FLS* and *UGT* in the flavonoid pathway are significantly higher in browning callus than in green callus.	[60]
	*LAR1*	*M. domestica*	*MdLAR1* inhibits the expression of other genes in the anthocyanin biosynthesis.	[61]
	*MYB21*, *MYB54*	*Pyrus ussuriensis* Maxim.	*PuMYB21/PuMYB54* enhance the degradation of membrane phospholipids leading to pericarp browning.	[62]
	*PAL*, *4CL*	*Pyrus spp*	Down-regulated expression of *PuPAL* and *Pu4CL* result in browning.	[63]
	*PAL*, *4CL*, *F3H*, *CYP73A*, *CHS*, *CHI*, *ANS*, *DFR*, *PGT1*	*Malus sieversii*	Genes related to flavonoid biosynthesis increase flavonoid accumulation during browning.	[64]
	*PAL*, *C4H*, *4CL*, *CCR*, *CAD*	*M. domestica*	Genes increase the biosynthesis of phenolic compounds contributing to browning.	[65]
	*PAL*, *ANS*	*J. regia*	These genes are involved in the synthesis of phenolic compounds and color changes in walnuts.	[66]

POD, a class of single-electron oxidizing enzymes, is prevalent in plants, animals, and microorganisms, and acts as a crucial endogenous ROS scavenger in cells^[54]^. Pang et al.^[55]^ demonstrated a synergistic effect between the cytokinin N-phenyl-N-thiazolylurea (PBU) and 6-benzylaminopurine (6-BA), which not only enhanced POD activity but also reduced callus browning of *Eucalyptus urophylla*, and promoted embryonic callus differentiation. This study also found that PBU and 6-BA have differential effects on the expression of POD isozymes. In *Arabidopsis thaliana*, transcription factors *BREVIPEDICELLUS* (*KNAT1/BP*) and *ERECTA* (*ER*) directly binds to the *Arabidopsis* *POD* (*AtPRX17*) promoter, supporting normal growth and browning control in callus by scavenging H_2_O_2_^[56]^. Interestingly, PPO and POD may act synergistically in the oxidation of phenolics, as H_2_O_2_ produced by PPO enhances POD activity^[57]^. Additionally, research on the browning in apple pericarp and callus has revealed the role of laccase (LAC) enzyme^[7]^; especially, the MdLAC7 protein catalyzes the oxidation of catechin and other phenolic acids such as vanillic acid, anthocyanin, tannic acid, and erucic acid, leading to browning^[58]^ (Table 1).

#### Phenylpropanoid pathway

**Figure 1:**
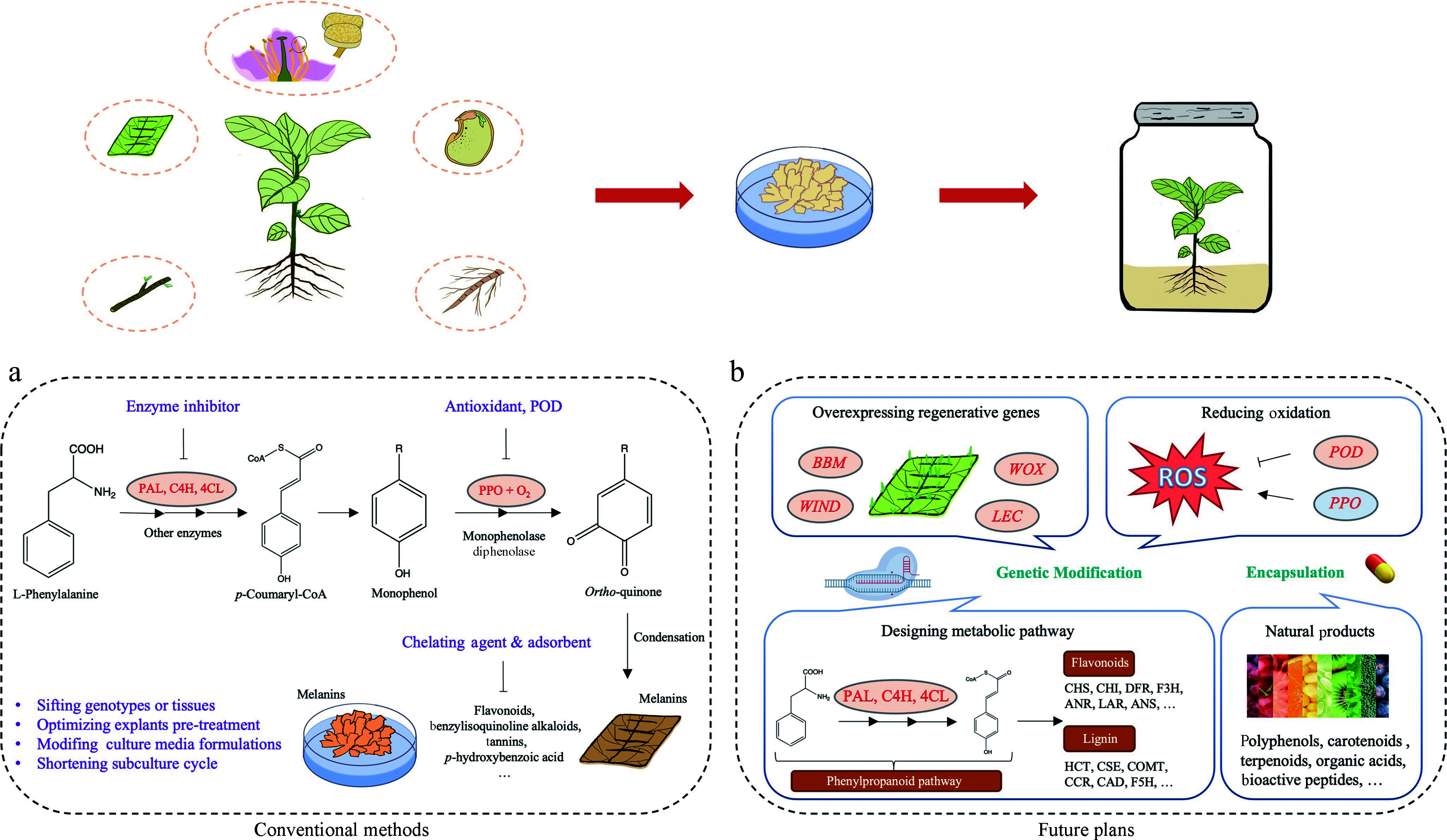
Techniques and perspectives on controlling browning in *in vitro* cultures of woody plants. (a) Traditional management strategies for controlling enzymatic browning. (b) Prospective strategies for addressing browning: genetic approaches by targeting key metabolic pathways and regenerative genes, encapsulation of natural products.

Plant phenolics, such as phenylpropanes, flavonoids, and tannins, are predominantly synthesized *via* the phenylpropanoid pathway. This pathway commences with the conversion of phenylalanine, which is produced *via* the shikimate synthesis pathway, into *p*-coumaroyl coenzyme A. This conversion involves the enzymes phenylalanine ammonia-lyase (PAL), cinnamic acid 4-hydroxylase (C4H) and 4-coumarate-CoA ligase (4CL)^[67]^. The *p*-Coumaroyl-coenzyme A then serves as a precursor for subsequent transformations into lignin and flavonoid synthesis pathway (Fig. 1a). Phenylalanine can be converted to free phenolic substrates for POD production through the catalytic activity of PAL, which also promotes PPO synthesis and enhances healing tissue browning^[68,69]^. Various studies have highlighted the pivotal role of genes related to the phenylpropanoid in regulating browning (Table 1). For instance, inhibiting PAL activity has demonstrated a reduction in browning in *Acer saccharum* and *Ulmus americana*^[70]^. Similarly, the expression of *leucoanthocyanidin reductase* (*LAR*) and *anthocyanidin reductase* (*ANR*) genes have been linked to browning in apple pulp through the biosynthesis of catechins and epicatechins^[61]^. Further studies have implicated genes like *chalcone synthase* (*CHS*), *dihydroflavanol 4-reductase* (*DFR*), *anthocyanidin synthase* (*ANS*), *flavonol synthase* (*FLS*), *Bronze 1* (*BZ1*), *bHLH35*, and *bHLH63* in the color formation of poplar leaves *via* the flavonoid metabolism pathway^[71]^. Transcriptomes and metabolomics comparison of green and browning callus in Ginkgo have shown that the accumulation of flavonoid glycosides is primarily controlled by flavonol synthase (*FLS*) and UDP-glucurono-syltransferase (*UGT*) genes^[60]^.

## Technological and methodological advances in mitigating browning

Enzymatic browning is the primary type of browning that occurs in exosomes which requires the simultaneous presence of substrates, enzymes, and oxygen^[72]^. Therefore, callus browning can be prevented by removing oxidizing substances, capturing or reducing intermediates in the polymerization reaction, and inhibiting the relevant enzyme activity. Nevertheless, callus browning is frequently influenced by various factors. Numerous methods exist to prevent browning, each with specific applicability and limitations. Consequently, addressing callus browning requires adopting appropriate measures to inhibit or mitigate browning effectively.

### Evaluating browning in tissue cultures

**Figure 2:**
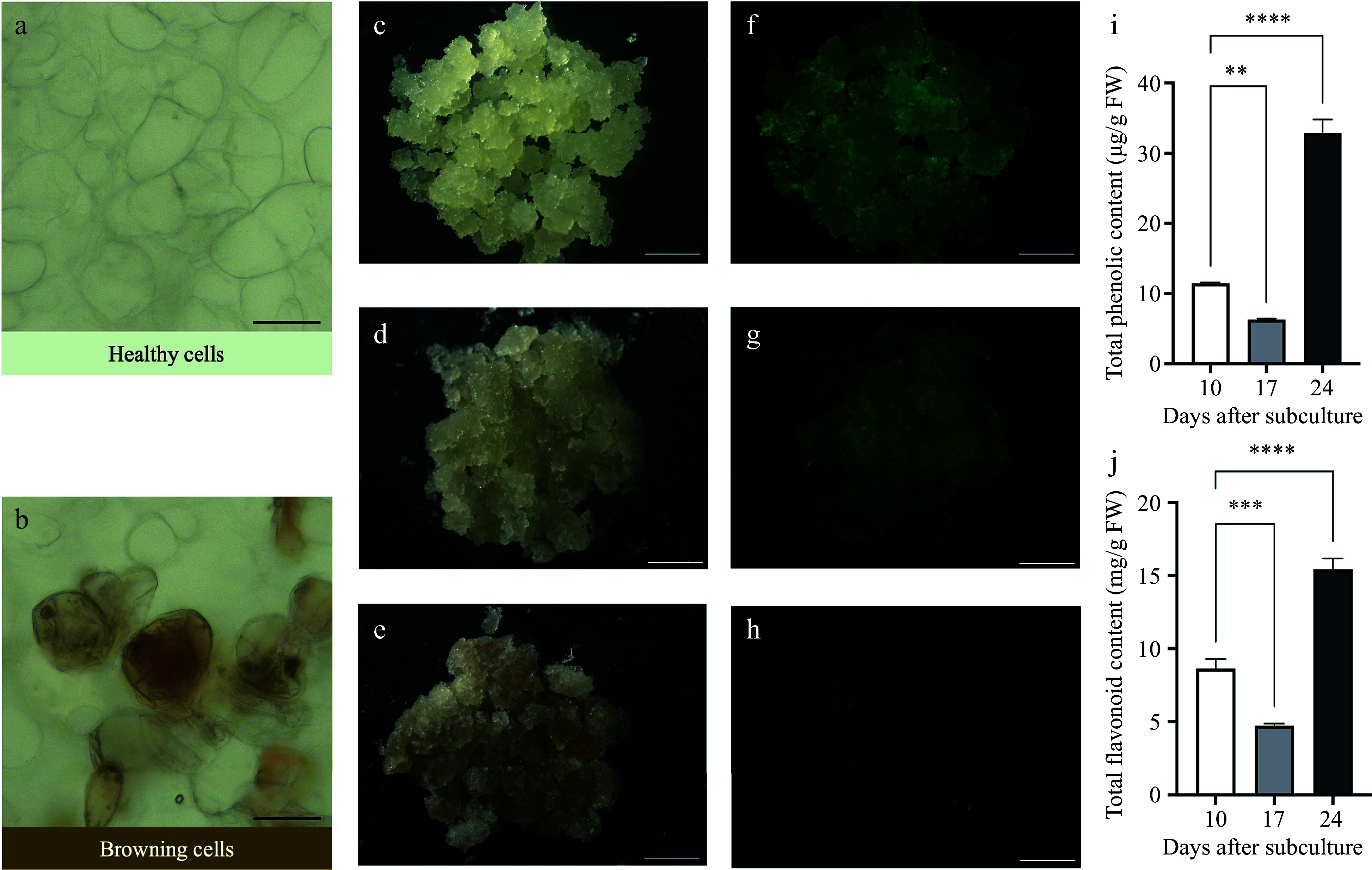
Comparison of healthy and browning callus in *Carya cathayensis* Sarg. Microscopic view of (a) healthy cells and (b) browning cells. Scale bars = 50 μm. Phenotypic progression of callus after subculture: (c) non-browning callus at day 10, (d) slightly browning callus at day 17, and (e) browning callus at day 24. Scale bars = 4 mm. FDA staining indicating viability of (f) non-browning callus, (g) slightly browning callus, and (h) browning callus. Scale bars = 4 mm. (i)−(j) Total phenolic content and total flavonoid content of non-browning callus at day 10, slightly browning callus at day 17, and browning callus at day 24. Error bars represent the SD (n = 3). Statistical analysis was performed using one-way ANOVA for each treatment (** *p* < 0.01, *** *p* < 0.001, **** *p* < 0.0001).

The presence of browning in callus can be discerned through its appearance and morphology. Normal callus exhibits well-organized and densely packed cells with growth potential. In contrast, browning callus displays disorganized, loosely distributed with a darker color and signs of ruptured cells, indicating a tendency towards cell death^[73]^. However, the morphology of normal or brown callus varies considerably between different explants. For instance, in Ginkgo, normal callus cells contain more starch and lipid droplets, while brown callus cells are richer in tannins^[60]^. In hickory, browning callus cells exhibited increased browning components within the cytoplasm and cell shrinkage, whereas normal callus cells were active in cell division (Fig. 2a & b).

Browning can be prevented by evaluating the health status of callus in advance. Conventional methods rely heavily on visual coloration, which is prone to subjective biases and can be compromised by chlorophyll masking the brown pigments. This highlights the necessity for standardized assessment techniques that encompass cell viability, division rate, differentiation capabilities, as well as morphological and cytological criteria to systematically evaluate browning levels. Fluorescein diacetate (FDA), an esterase substrate permeable to the cell membrane, has proven effective for analyzing cell viability. In our study on hickory, we utilized FDA to track the viability changes of callus after subculture (Fig. 2c−h). After ten days of subculture, the callus exhibited a white or light-yellow color, characterized by rapid proliferation and intense fluorescence (Fig. 2c & f), along with relatively low levels of total phenolic and flavonoid contents (Fig. 2i & j). By day 17, the callus proliferation diminished, fluorescence intensity decreased (Fig. 2d & g), and browning increased, accompanied by a lower accumulation of total phenolic and flavonoid contents (Fig. 2i & j). By day 24, the callus showed extensive browning, minimal fluorescence (Fig. 2e & h), and significantly elevated levels of total phenolic and flavonoid contents (Fig. 2i & j). The FDA staining method not only indicates browning similar to visual evaluation but is more sensitive to subtle color changes, reducing biases among different researchers. Moreover, it effectively monitors the progression of browning, helping researchers to implement suitable interventions as necessary, offering enhanced convenience and adaptability.

In conclusion, our study demonstrates the limitations of traditional visual assessments for browning and confirm the efficacy of FDA as a precise and quantifiable indicator of cell viability in hickory callus cultures. This substantiates the need for standardized methods that integrate FDA analysis to enhance accuracy in monitoring and optimizing tissue culture conditions.

### Optimizing pre-treatment and culture conditions to prevent browning

Traditionally, preventing browning in tissue culture involves the application of various browning inhibitors that target specific stages of the browning process. There are three main types of reagents commonly employed to mitigate browning in tissue culture: antioxidants, adsorbents, and competitive inhibitors.

#### Antioxidants

Antioxidants, including ascorbic acid (VC)^[74]^, citric acid^[75]^, glutathione (GSH)^[76]^, L-cysteine^[77]^, mannitol^[78]^, and silver nitrate (AgNO_3_)^[79]^, and sodium thiosulfate (Na_2_S_2_O_3_)^[80]^, play critical roles in improving the intracellular redox environment. They protect against oxidative stress that damage cellular structure and maintain phenolic homeostasis. 1.0 g/L ascorbic acid significantly inhibits explants browning in *Sapindus mukorossi*^[74]^, while 0.1 g/L ascorbic acid or 0.2 g/L L-cysteine is sufficient in the callus of *Cunninghamia lanceolata*^[77]^. Immersing barberry (*Berberis integerrima*) explants in a 0.3 g/L citric acid solution for 30 min and supplementing the culture medium with 0.225 g/L of citric acid efficiently manage browning^[75]^. Treating pistachio (*Pistacia vera*) shoot tips with a 0.1 mM GSH solution before further processing is also beneficial^[76]^. Additionally, controlling grape (*Vitis vinifera*) callus browning can be achieved with 2.0 g/L PVP, 20 g/L mannitol, or 0.02 g/L AgNO_3_^[78]^.

#### Adsorbents

Adsorbents like polyvinylpyrrolidone (PVP)^[81]^, and charcoal^[82]^ absorb phenolic compounds that contribute to browning. PVP, available in various molecular weights, requires careful selection to ensure efficacy^[78]^. Charcoal operates through intermolecular hydrogen bonding and van der Waals forces to absorb substances that cause browning^[83]^. A 1 g/L PVP treatment can reduce the production of phenolic compounds and promote callus regeneration in tree peony (*Paeonia suffruticosa* Andrews)^[81]^. Charcoal is often used in conjunction with other browning inhibitors. For instance, a combination of 1.0 g/L activated carbon (AC) and 30 g/L Na_2_S_2_O_3_ effectively inhibit browning in *Prunus avium*^[80]^. In addition, date stone-based activated carbon (DSAC) is an efficient natural anti-browning compound for date palm organogenesis. A concentration of 1.5 g/L DSAC significantly improves shoot bud proliferation and reduces tissue browning in *Phoenix dactylifera*^[82]^.

#### Competitive inhibitors

Disodium ethylenediaminetetraacetic acid (EDTA) acts as a competitive inhibitor capable of binding to polyphenol oxidase, thereby reducing phenol oxidation^[40]^. Although EDTA's broad chelation properties limit its use *in vitro*, it illustrates the specificity of chelators in managing phenolic reactions. Enzyme inhibitors like aminoindane-2-phosphonic acid (AIP) effectively inhibit the activity of PAL and is added to media to limit substrate-enzyme interactions and mitigate browning^[70]^. In addition, epigallocatechin gallate (EGCG) exhibits high inhibition towards PPO, making it a potential browning inhibitor^[45]^.

These browning inhibitors can be used individually or in combination, either by presoaking explants or adding them directly to the culture medium. However, due to the inherent variability among plant species, no single method of using browning inhibitors is universally effective for all plants.

### Genetic approaches by targeting key metabolic pathways and retentive genes

Although some browning inhibitors can mitigate the effect of browning on explants, they typically address the symptoms rather than the underlying cause, particularly in plants prone to browning. To fundamentally resolve the browning issue, further research is required to elucidate the molecular mechanisms underlying this phenomenon. Increasing attention is focusing on the novel functions of genes related to redox and phenolics metabolism, which play crucial roles in regulating browning in tissue-cultured materials. Genetic modification tools have been applied to develop varieties less prone to browning by targeting these specific pathways (Fig. 1b).

#### Oxidative-related genes

Manipulating the gene expression of *PPOs* and *PODs* in callus can inhibit their browning. Transcriptomic analysis by Deng et al. found that the expression of two *PPOs* and 17 *PODs* is significantly upregulated during the browning process in lotus callus^[84]^. In addition, Pompili et al*.* discovered that overexpressing *PPO6*, *PPO7*, and *PPO11* in the callus of globe artichoke (*Cynara cardunculus* var. *scolymus* L.) lead to browning phenotypes due to phenol oxidation^[85]^. In *Arabidopsis*, *AtPRX17* maintains rapid callus growth without promoting callus induction and is regulated by BREVIPEDICELLUS (KNAT1/BP) and ERECTA (ER), which inhibit callus browning^[56]^. Similarly, in apples, knockout *MdPPO1* *via* CRISPR/Cas9 successfully reduces browning^[86]^. Zhang et al.^[87]^ identified *BROWNING OF CALLUS1* (*BOC1*) in indica rice (*Oryza sativa* L.), which encodes a SIMILAR TO RADICAL-INDUCED CELL DEATH ONE (SRO) protein. Up-regulating *BOC1* reduces callus browning and improves the genetic transformation efficiency, suggesting its role in mitigating oxidative stress-induced cell senescence and death.

#### Genes in the phenylpropanoid pathway

Phenolics that promote enzymatic browning are mainly derived from the phenylpropanoid pathway, which controls the biosynthesis of flavonoids and lignans^[88]^. Discovering the genes related to the phenylpropanoid pathway and then manipulating phenolic synthesis through molecular biology technology can alleviate or inhibit callus browning. In recent years, researchers have found that genes such as *PALs*, *HCTs* (hydroxycinnamoyl transferases), *CHIs* (chalcone isomerases) and other related genes are more highly expressed in brown callus compared to normal callus through fluorescence quantitative PCR and transcriptome analysis^[60,69]^, suggesting that these genes can promote callus browning. Additionally, Wei et al.^[89]^ transformed the *JsFLS5* (flavonol synthetase) gene into the leaf-derived callus of 'Qianhe-7', resulting in improved content of total flavonoids, implying that *JsFLS5* inhibits the browning of walnut callus. In poplar, overexpressing *PtrMYB119* or *PtrMYB120* shows elevated accumulation of cyanidin-3-O-glucoside, with upregulation of *PtrCHS1* (chalcone synthase1) and *PtrANS2* (anthocyanin synthase2)^[90]^.

#### Regenerative genes

Utilizing a range of regeneration-promoting genes can significantly enhance the regeneration efficiency in woody plants, effectively addressing browning issues (Table 2). These genes include those involved in hormone signaling, cell differentiation, growth, and regeneration. Duan et al.^[91]^ identified *CYTOKININ OXIDASE/DEHYDROGENASE 9* (*OsCKX9*) as a key strigolatone-responsive gene. Overexpression of *OsCKX9* upregulates rice *type-A response regulator1* (*OsRR1*) and *OsRR2*, reducing total cytokinin content and decreasing browning. Phytosulfokine (PSK), a plant peptide hormone, promotes somatic embryogenesis in *C. lanceolata*. When heterologously expressed in *Arabidopsis*, *ClPSK* promotes root growth by maintaining H_2_O_2_ homeostasis^[92]^. Furthermore, the Wuschel-like homeobox protein (WOX) 13 alters the cell wall properties to enhance efficient callus formation and organ reattachment^[93]^. The conserved wound signal molecule Regeneration factor1 (REF1) boosts regeneration and genetic transformation efficiency in tomatoes, overcoming species and genotype limitations^[94]^. *WOX2a* in maize facilitates embryogenic callus and somatic embryo formation, leading to the regeneration of phenotypically normal plants and progeny (*Zea mays*)^[95]^. Similar functions are also observed in *Pinus pinaster*^[96]^, *Picea abies*^[97]^, and *C. lanceolata*^[98]^.

**Table 2:** Regeneration-promoting genes used in plant genetic transformation.

Gene	Species	Organ	Function	Ref.
*ATXR2*	*A. thaliana*	Callus, root	*ATXR2* promotes callus formation and lateral root growth	[99]
*BBM*	*Saccharum officinarum* L.	Callus	BBM exhibits high levels of expression in embryogenic callus	[100]
*CKI*	*Gossypium hirsutum*	Hypocotyl section	Overexpression of *GhCKI* inhibits somatic embryo formation and plant regeneration	[101]
*GRF4-GIF1*	*Triticum aestivum*		*GRF4-GIF1* improves regeneration efficiency and overcomes genotypic limitation	[102]
*IAA*	*Moringa oleifera* Lam.	Shoot	*IAA13* inhibits shoot regeneration	[103]
*LBD5*	*Z. mays*		Overexpression of *ZmLBD5* in *Arabidopsis* reduces ROS	[104]
*LBD19*	*A. thaliana*	Callus	Negatively regulates callus formation	[105]
*LEC*	*S. officinarum* L.	Callus	*LEC* exhibits high levels of expression in embryogenic callus	[100]
*PSK*	*C. lanceolata*	Somatic embryogenesis	Overexpression of *ClPSK* in *Arabidopsis* enhances somatic embryogenesis capabilities and lowers hydrogen peroxide levels	[92]
*REF1*	*Solanum lycopersicum* L.	Callus, shoot	Upon cellular damage, *REF1* serves as an initial wound signal molecule and is recognized by the receptor *PORK1*, activating the expression of key cell reprogramming regulator *SlWIND1*	[94]
*WIND*	*A. thaliana*	Callus, shoot	*WIND1* promotes callus formation and shoot regeneration	[106]
*WOX*	*Salix suchowensis*	Shoot	*WOX* promotes shoot regeneration	[107]
*WOX2*	*P. abies*	Somatic embryos	Down-regulation of *PaWOX2* early in embryo development significantly decreases in the yield of mature embryos	[97]
	*P. pinaster*	Callus	*WOX2* serves as a marker gene for somatic embryogenesis	[96]
*WOX5*	*T. aestivum*	Callus	*WOX5* increases transformation efficiency with less genotype dependency	[108]
	*C. lanceolata*	Leaf	Overexpression of *WOX5* improves shoot regeneration but causes aborted embryo development, resulting in a partially sterile phenotype	[98]
*WOX6*	*C. lanceolata*	Leaf	Overexpression of *WOX6* improves shoot regeneration	[98]
*WOX11*	'84K' (*P. alba* × *P. glandulosa*)	Leaf	*PtWOX11* promotes de novo root, shoot organogenesis in poplar	[109]
*WOX13-1*	*Malus × domestica*	Callus	*MdWOX13-1* increases callus weight and enhanced ROS scavenging ability	[110]

These genetic modifications not only accelerate cell proliferation and promote cell metabolism but also rapidly consume or reduce the accumulation of browning substances, thereby alleviating the browning.

### Encapsulation of natural products

Recent advances have increasingly focused on the utilization of natural products derived from botanical sources for postharvest preservation^[111]^. These natural products include polyphenols^[112]^, carotenoids^[113]^, terpenoids^[114]^, organic acids^[115]^ and bioactive peptides^[116]^. However, maintaining the efficacy of these active compounds is challenging due to their susceptibility to degradation from oxygen and humidity. One method, known as encapsulation, has been developed to protect these bioactive substances from degradation and oxidation, ensuring sustained efficacy throughout the culture process^[111]^. Encapsulation of natural products is a novel and promising strategy for mitigating browning in plant tissue cultures. This technique encases bioactive compounds, such as antioxidants and anti-browning agents, within protective matrices to enhance their stability and facilitating controlled release^[117]^. Such encapsulation has been shown to significantly reduce browning due to their antioxidant properties and ability to inhibit enzymatic activities responsible for browning (Fig. 1b).

## Prospects

This review provides an overview of the predominant strategies to mitigate tissue culture browning and explores potential future directions, such as the application of genetic modification techniques and natural products. However, certain obstacles must be addressed to advance this field.

Numerous key genes are involved in the metabolic pathways of phenolic compounds, but their roles in browning, whether positive or negative, remains unclear. Comprehensive and systematic research on the browning regulation network, utilizing high-throughput sequencing technologies, is needed. Additionally, key genes in metabolic pathways and regenerative genes variably affect the development and growth of different species. Identifying regenerative-related marker genes common in most plants is crucial. By regulating these maker genes' expression, browning in tissue culture can be significantly reduced, leading to superior varieties.

Innovations in genetic transformation are crucial for future research. Traditional methods like *Agrobacterium* and gene guns often damage plant materials, leading to tissue culture browning. Novel and optimized transgenic approaches hold promise for reducing browning and enhancing transformation efficiency. For instance, carbon nanomaterials are increasingly favored for their small size and minimal damage to plants. These novel transformation vectors could become mainstream technologies in the future.

While natural products are widely used in post-harvest storage, they are less applied in plant tissue culture. Further screening of natural products for culture media is needed, and their efficient utilization must be optimized to prevent ineffectiveness. In conclusion, future trends in tissue culture may involve the discovery of natural products with strong anti-browning capabilities, ease of use, and long-lasting effectiveness.

## Conclusions

Browning significantly impedes the regeneration and genetic transformation of economically important woody plants. Extensive research into browning has encompassed physiological, biochemical, and advanced molecular dimensions. However, traditional methods of browning assessment based on visual evaluation are inadequate. The present findings underscore the effectiveness of FDA staining as a reliable, quantitative measure of cell viability.

By deepening our understanding of the pathways and regulatory mechanisms involved in browning and integrating advanced strategies such as genetic manipulation and natural product encapsulation, we can develop more targeted approaches to control browning in tissue cultures. This will ultimately enhance the efficiency of plant regeneration technologies and significantly contribute to advancements in plant tissue culture and the broader field of plant biotechnology. Moreover, by strategically redirecting its metabolic flux, we can mitigate browning without disrupting the production of essential metabolites.

## Author contributions

The authors confirm contribution to the paper as follows: conception and design: Zhou X, Cao F; data collection: Fan H, Wu J; data analysis and manuscript preparation: Liu C, Fan H, Zhang J; manuscript revision: Tao G, Liu C, Zhou X. All authors reviewed the results and approved the final version of the manuscript.

## Data availability

All data generated or analyzed during this study are included in this published article.
